# Homotypic protection against influenza in a pediatric cohort in Managua, Nicaragua

**DOI:** 10.1038/s41467-022-28858-9

**Published:** 2022-03-04

**Authors:** Steph Wraith, Angel Balmaseda, Fausto Andres Bustos Carrillo, Guillermina Kuan, John Huddleston, John Kubale, Roger Lopez, Sergio Ojeda, Amy Schiller, Brenda Lopez, Nery Sanchez, Richard Webby, Martha I. Nelson, Eva Harris, Aubree Gordon

**Affiliations:** 1 Department of Epidemiology, School of Public Health, University of Michigan, Ann Arbor, MI USA; 2 Sustainable Sciences Institute, Managua, Nicaragua; 3 Laboratorio Nacional de Virología, Centro Nacional de Diagnóstico y Referencia, Ministry of Health, Managua, Nicaragua; 4 Division of Infectious Diseases and Vaccinology, School of Public Health, University of California, Berkeley, Berkeley, CA USA; 5 Centro de Salud Sócrates Flores Vivas, Ministry of Health, Managua, Nicaragua; 6 Vaccine and Infectious Disease Division, Fred Hutchinson Cancer Research Center, Seattle, WA USA; 7 St. Jude Children’s Research Hospital, Memphis, TN USA; 8 National Institutes of Health, Bethesda, MD USA

**Keywords:** Influenza virus, Viral infection, Epidemiology

## Abstract

The period of protection from repeat infection following symptomatic influenza is not well established due to limited availability of longitudinal data. Using data from a pediatric cohort in Managua, Nicaragua, we examine the effects of natural influenza virus infection on subsequent infection with the same influenza virus subtype/lineage across multiple seasons, totaling 2,170 RT-PCR-confirmed symptomatic influenza infections. Logistic regression models assessed whether infection in the prior influenza season protected against homologous reinfection. We sequenced viruses from 2011–2019 identifying dominant clades and measuring antigenic distances between hemagglutinin clades. We observe homotypic protection from repeat infection in children infected with influenza A/H1N1pdm (OR 0.12, CI 0.02–0.88), A/H3N2 (OR 0.41, CI 0.24–0.73), and B/Victoria (OR 0.00, CI 0.00–0.14), but not with B/Yamagata viruses (OR 0.60, CI 0.09–2.10). Overall, protection wanes as time or antigenic distance increases. Individuals infected with one subtype or lineage of influenza virus have significantly lower odds of homologous reinfection for the following one to two years; after two years this protection wanes. This protection is demonstrated across multiple seasons, subtypes, and lineages among children.

## Abstract (web summary)

Here Wraith *et al*. report homotypic protection from repeated influenza infection in a prospective pediatric cohort in Nicaragua followed for 9 years. This protection is observed across multiple seasons, subtypes, and lineages and is consistent for older and younger children.

## Introduction

Influenza poses a significant public health threat, with the effects of repeated infections still not well understood^1,2^. Children experience particular influenza risk and high attack rates during outbreaks^3^. The effects of homotypic influenza protection have been explored previously but rarely in longitudinal studies^4–11^. Several important studies have previously examined influenza infection patterns in a range of contexts, and with this study, we seek to build upon this historical work. The Tecumseh Study examined illness duration and the age-related factors driving this from 1965 to 1972 and 1976 to 1981, identifying age-specific infection rates and peak ages for influenza A and B infection^12^. The Cleveland and Houston family cohorts both conducted household-based studies to track outbreaks and characterize the incidence and noted the presence of protection from re-infection in some seasons but not others^13–15^. While these historical studies laid important groundwork for the examination of homotypic protection, they were limited in both power and by the laboratory techniques of the time, which restricted the ability to time infection events precisely. More recently, a range of cohort studies has been conducted in both temperate and tropical contexts to explore influenza burden and measure immune responses. The Flu Watch cohort in the UK demonstrated evidence that T-cell-based immunity provided protection from influenza infection in both seasonal and pandemic periods^16,17^. In children in Hong Kong, protection from reinfection for both A/H1N1pdm and H3N2 in one subsequent homologous season has been observed using serological samples and model simulation^18,19^. Other studies in Singapore and Vietnam have examined influenza dynamics and incidence but have not characterized the level of homotypic protection from symptomatic infection^20,21^. Additionally, no data exist showing protection from clinical infection or for multiple seasons which is crucial for influenza given varying antigenic change between seasons. Here we examine homotypic influenza protection in children utilizing natural symptomatic infections covering a much broader 9-year time span.

Immunity to influenza is driven by antibody responses to surface glycoproteins hemagglutinin (HA) and neuraminidase (NA). Antibody responses to infection drive evolutionary pressures and antigenic drift, necessitating the creation of yearly influenza vaccines. For the development of next-generation influenza vaccines, it is critical to determine the extent of immunity conferred by natural infection^22–26^. In this analysis, we explore interactions between patterns of virus drift and duration of time between infections, showing how these dynamics drive protection from symptomatic influenza infection.

## Results

Between January 2011 and December 2019, we followed 2764 participants aged 0–14 years who experienced 2170 episodes of symptomatic, reverse transcription-polymerase chain reaction (RT-PCR)-confirmed influenza. We identified 542A/H1N1pdm infections, 867A/H3N2 infections, and 798 influenza B infections, with 37 infections being co-infections.

**Fig. 1:**
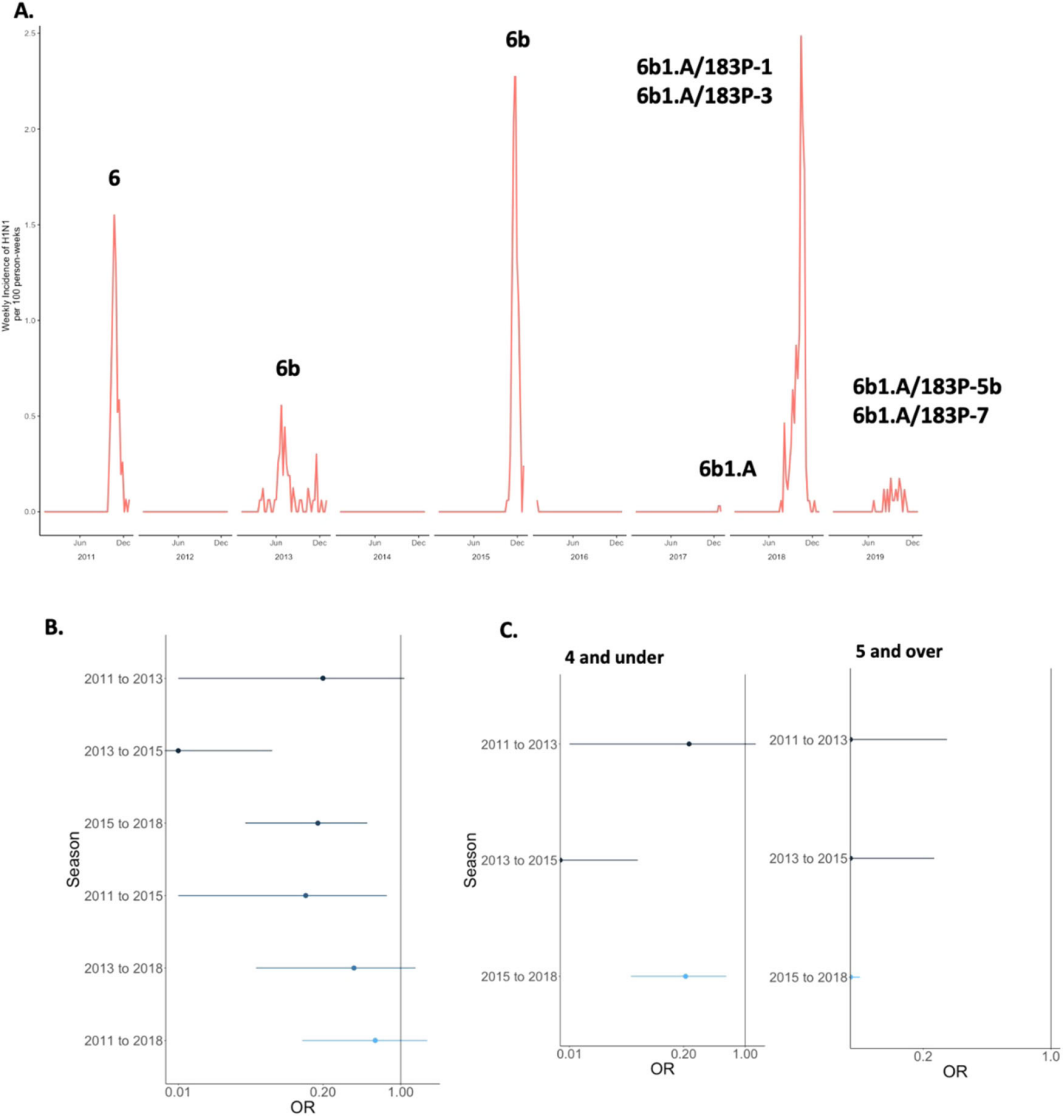
Influenza A/H1N1pdm epidemics and duration of protection across seasons. **A** Seasonality and clades of influenza A H1N1pdm cases among children in the study from 2011 to 2019. **B** Log-adjusted odds ratios for a given exposure and outcome year looking at protection from repeat infection with error bars for the confidence intervals. **C** Log-adjusted odds ratios for a given exposure and outcome year looking at protection from repeat infection, stratified on age and restricted to season distances less than 4 years, with error bars for the confidence intervals. For **B**, **C**, *n* = 2764 children followed over 9 study years. Darker colors in the plots represent seasons that are closer together in time; lighter colors represent seasons that are further apart.

**Fig. 2:**
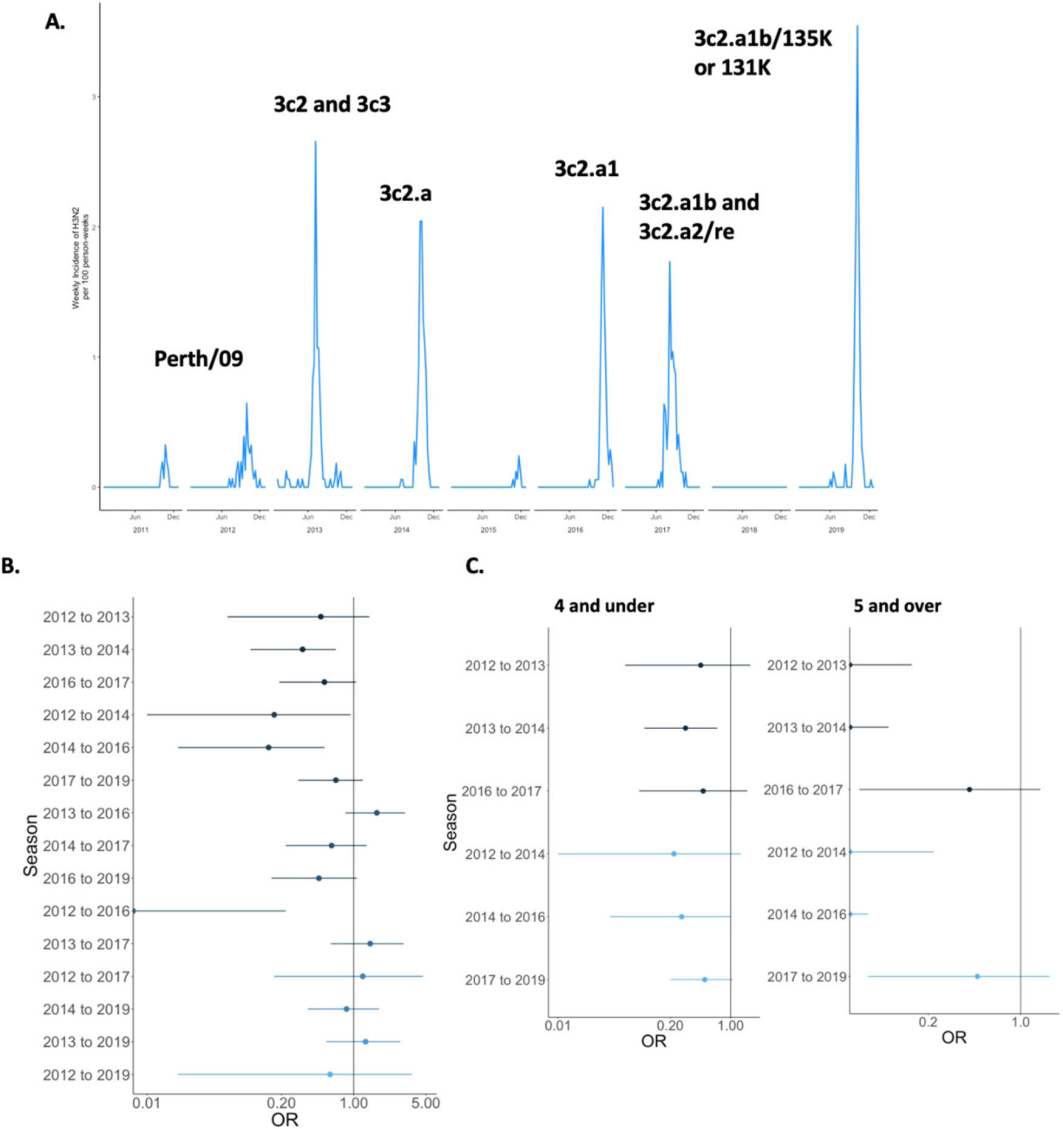
Influenza A/H3N2 epidemics and duration of protection across seasons. **A** Seasonality and clades of influenza A H3N2 cases among children in the study from 2011 to 2019. **B** Log-adjusted odds ratios for a given exposure and outcome year looking at protection from repeat infection with error bars for the confidence intervals. **C** Log-adjusted odds ratios for a given exposure and outcome year looking at protection from repeat infection, stratified on age and restricted to season distances less than 4 years, with error bars for the confidence intervals. For **B**, **C**, *n* = 2764 children followed over 9 study years. Darker colors in the plots represent seasons that are closer together in time; lighter colors represent seasons that are further apart.

**Fig. 3:**
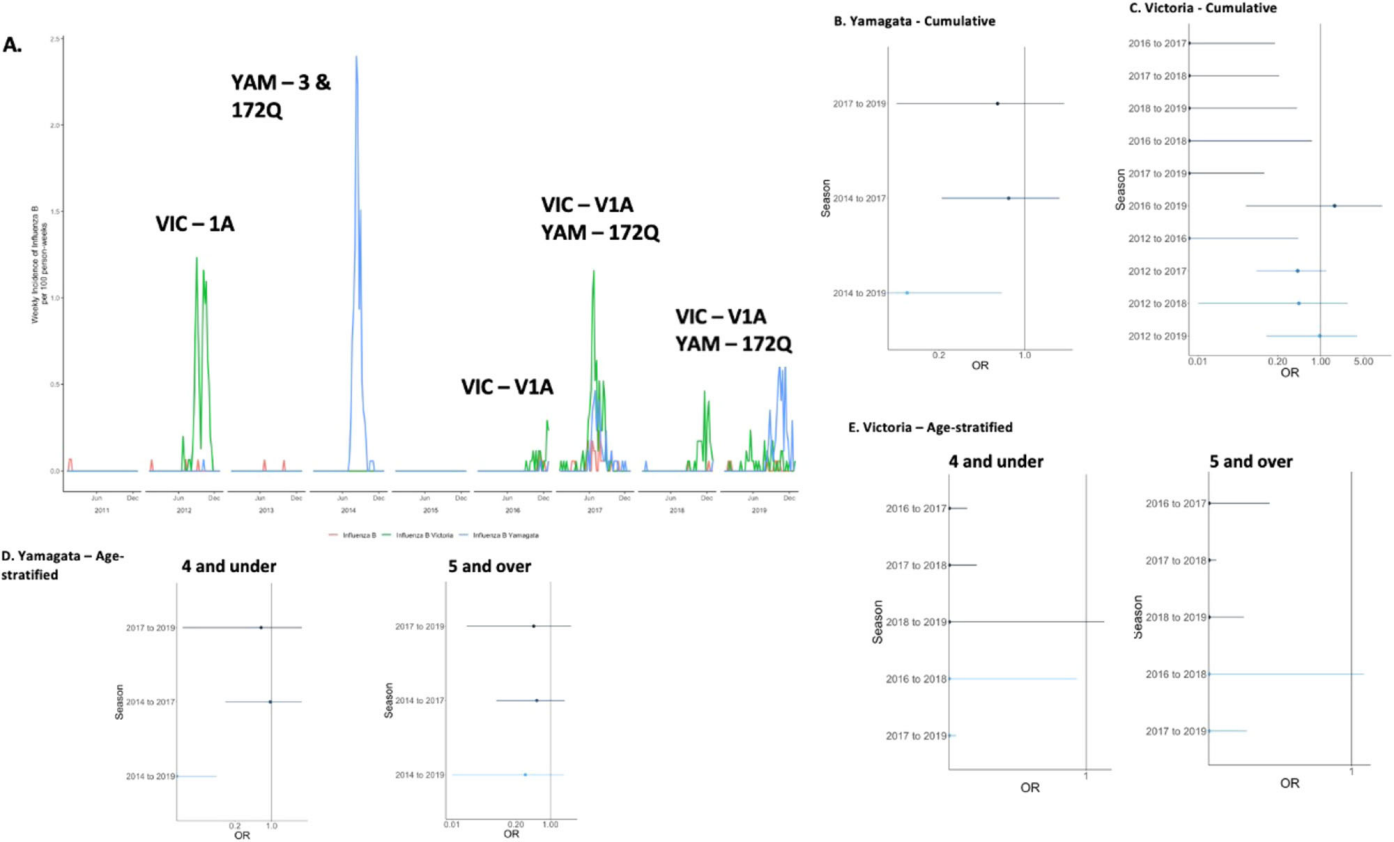
Influenza B epidemics and duration of protection across seasons. **A** Seasonality and clades of influenza B cases among children in the study from 2011 to 2019. **B** Log-adjusted odds ratios for a given exposure and outcome year looking at protection from repeat infection with Yamagata lineage, with error bars for the confidence intervals. **C** Log-adjusted odds ratios for a given exposure and outcome year looking at protection from repeat infection with Victoria lineage, with error bars for the confidence intervals. **D** Log-adjusted odds ratios for a given exposure and outcome year looking at protection from repeat infection with Yamagata lineage, stratified on age and restricted to season distances less than 4 years, with error bars for the confidence intervals. **E** Log-adjusted odds ratios for a given exposure and outcome year looking at protection from repeat infection with Victoria lineage, stratified on age and restricted to season distances less than 4 years, with error bars for the confidence intervals. For **B**–**E**, *n* = 2764 children followed over 9 study years. Darker colors in the plots represent seasons that are closer together in time; lighter colors represent seasons that are further apart.

The dominant influenza A subtype fluctuated from year to year. A/H1N1pdm dominated in 2011, 2015, and 2018, and there were few if any cases in 2012, 2014, 2016, and 2017 [Table S1]. A/H3N2 was the dominant influenza A subtype in 2012–2014, 2016–2017, and 2019. Influenza B lineages fluctuated between B/Yamagata and B/Victoria, with B/Victoria being more dominant in recent years when lineage typing was routinely available. The subtypes and lineages underwent highly varied levels of antigenic drift over the study period [Figs. 1–3]. A/H1N1pdm viruses remained fairly stable except for a shift from genetic clade 6 to 6b in 2013 and a subsequent shift to 6b.1a in 2017. A/H3N2 viruses underwent more evolution, with a different genetic clade dominating circulation for each season after 2012. For influenza B, the B/Victoria lineage clade was very stable throughout 2012–2017, while more genetic change occurred in B/Yamagata during 2014–2017.

Overall, A/H1N1pdm exhibited very strongly protective effects against homotypic infection, with an overall subsequent season protection aOR of 0.12 (CI: 0.02–0.88) [Table 1]. With the exception of being infected in 2011 and experiencing a symptomatic infection in 2013, all seasons ≤4 years apart exhibited strong protection from repeated symptomatic infection. The strongest protection occurred for A/H1N1pdm cases in 2013, whose aOR for repeated symptomatic infection in 2015 compared to those who were not infected in 2013 was 0.01 (CI: 0.00–0.07) [Table 1, Fig. 1]. A/H1N1pdm cases from 2011 had a similar level of protection in 2015 (aOR: 0.14, CI: 0.01-0.75), as did individuals infected in 2015 against illness in 2018 (aOR: 0.18, CI: 0.04–0.50). Prior symptomatic A/H1N1pdm infection protected from repeated symptomatic infection equivalent to vaccine effectiveness of 80–99% over two years and 86% over four years. For seasons five or more years apart, no significant protection was observed. The antigenic distances between all seasons were <0.5 on the log_2_ scale, indicating relative stability over the study period. There was a shift from clade 6 to 6a from 2011 to 2013 that may explain some of the diminished protection during that time period [Fig. S2].

**Table 1:** Influenza A repeat infection odds.

Seasons	*N*	NRI	aOR*	95% CI	Antigenic distance
A/H1N1pdm					
*Two years apart*					
Summary		1	0.12	(0.02–0.88)	
2011–2013	1468	1	0.20	(0.01–1.08)	^a^
2013–2015	1456	0	0.01	(0.00–0.07)	0.47
*Three years apart*					
2015–2018	1363	3	0.18	(0.04–0.50)	0.07
*Four years apart*					
2011–2015	1138	1	0.14	(0.01–0.75)	^a^
*Five years apart*					
2013–2018	885	2	0.38	(0.05–1.36)	0.07
*Seven years apart*					
2011–2018	659	3	0.59	(0.13–1.74)	^a^
A/H3N2					
*One year apart*					
Summary		13	0.41	(0.24–0.73)	
2012–2013	1566	2	0.48	(0.06–1.41)	0.3
2013–2014	1675	5	0.32	(0.10–0.67)	−0.6
2016–2017	1725	6	0.52	(0.19–1.05)	0.7
*Two years apart*					
Summary		12	0.41	(0.23–0.73)	
2012–2014	1335	1	0.17	(0.01–0.93)	1.3
2014–2016	1464	2	0.15	(0.02–0.52)	0.1
2017–2019	1586	9	0.67	(0.29–1.22)	2.1
*Three years apart*					
Summary		23	0.85	(0.55–1.33)	
Summary for post-2014		11	0.55	(0.30–1.03)	
2013–2016	1105	12	1.67	(0.83–3.13)	0.78
2014–2017	1227	6	0.61	(0.22–1.33)	0.06
2016–2019	1309	5	0.46	(0.16–1.07)	0.46
*Four years apart*					
2012–2016	864	0	0.00	(0.00–0.22)	0.5
2013–2017	907	8	1.44	(0.60–3.03)	0.9
*Five years apart*					
2012–2017	698	2	1.22	(0.17–4.65)	0.9
2014–2019	901	8	0.85	(0.36–1.75)	0.39
*Six years apart*					
2013–2019	632	8	1.30	(0.54–2.82)	0.78
*Seven years apart*					
2012–2019	462	1	0.59	(0.02–3.66)	^a^

^a^No antigenic distance is available between these seasons’ dominant clades.

*All models were adjusted for the age and sex of participants.

Upon stratifying A/H1N1pdm infections by age, patterns of protection persisted. There were some variations by age group: children aged ≥5 who were infected in 2011 were protected from repeat illness in 2013 (aOR: 0.00, CI: 0.00–0.27) as compared to children aged ≤4 who were not significantly protected (aOR: 0.23, CI: 0.01–1.32) [Table S2, Fig. 1]. Children aged ≥5 infected in 2011 were also significantly protected in 2018 (aOR: 0.00, CI: 0.00–0.22). Thus, while both age groups were well-protected from repeat symptomatic infections with A/H1N1pdm, older children (aged ≥5) at the time of infection were more strongly protected from subsequent homotypic A/H1N1pdm infection than younger children.

The H3N2 subtype of influenza A tended not to exhibit as strong a protective effect as H1N1pdm, particularly across two or more seasons. Overall, A/H3N2 had a 1-year protection aOR of 0.41 (CI: 0.24–0.73) and a near-identical 2-year protection aOR [Table 1]. However, unlike for A/H1N1pdm, we only observed A/H3N2-associated protection lasting for 4 years for a single season-to-season comparison; all other protective durations lasted 1 or 2 years. There was significant protection from repeated illness for 2012–2014, 2013–2014, and 2014–2016 [Table 1, Fig. 2]. For each of these associations apart from 2012 to 2014, antigenic distances between dominant clades in the given seasons were <0.4 on the log_2_ scale, indicating that circulating viruses at that time were similar. We were particularly interested in examining associations around the 2014 season, where a major clade shift took place in the circulating A/H3N2 virus, as we wanted to explore whether natural infection would still provide strong protection in the aftermath of a significant viral shift. Among children who were protected in 2014 as a result of an infection in 2013, we observed an increased risk of repeat infection in 2016 (aOR: 1.67, CI: 0.83–3.18) demonstrating the effect of the clade shift on homotypic protection, though this risk was not statistically significant. Additionally, when we examined the three-year protection from only clade-shifted A/H3N2 3c2.a viruses post-2014, we found borderline significant protection (aOR: 0.55, CI: 0.30–1.03), indicating that while protection from A/H3N2 among children generally lasts for a period of 2 years this protection may last up to 3 years if there is less antigenic drift in the virus, and reinforcing the joint impact of time and antigenic distance on protection from influenza.

When stratified by age, older children were better protected from repeated homotypic infection with A/H3N2 over mostly 1- and 2-year periods. Children aged ≥5 were significantly protected from 2012 to 2013, 2013 to 2014, 2012 to 2014, 2014 to 2016, and 2012 to 2016 (aOR: 0.00, CI: 0.00–0.26) [Table S3, Fig. 2]. However, children aged ≤4 infected in 2012 appeared to experience a longer duration of protection. Younger children infected in 2012 were not significantly protected from illness in 2013 or 2014 but were significantly protected from illness in 2016 (aOR: 0.00, CI: 0.00–0.35), 2017 (aOR: 0.00, CI: 0.00–0.33), and 2019 (aOR: 0.00, CI: 0.00–0.42); this discrepancy may be due to age-driven patterns of exposure and imprinting, or could be due to small number considerations. All results for Influenza A were similar in the sensitivity analyses conducted [Tables S4–S7].

The B/Victoria lineage circulated more frequently in the study area during 2011–2019 and was more prevalent in recent years, coinciding with greater availability of lineage typing and permitting more comparisons. Overall, there was strong homotypic protection against repeated symptomatic infection with the B/Victoria lineage for all seasons 1 or 2 years apart (1-year aOR 0.00 (CI: 0.00–0.28)) [Table 2, Fig. 3]. This pattern of short-term protection was consistent for both younger and older children [Table S8].

**Table 2:** Influenza B repeat infection odds.

Seasons	*N*	NRI	aOR*	95% CI	Antigenic distance
B/Victoria					
*One year apart*					
Summary		0	0.00	(0.00–0.14)	
2016 to 2017	1725	0	0.00	(0.00–0.18)	1.6
2017 to 2018	1727	0	0.00	(0.00–0.21)	1.6
2018 to 2019	1730	0	0.00	(0.00–0.41)	1.6
*Two years apart*					
Summary		0	0.00	(0.00–0.32)	
2016 to 2018	1457	0	0.00	(0.00–0.72)	1.6
2017 to 2019	1550	0	0.00	(0.00–0.12)	1.6
*Three years apart*					
2016 to 2019	1295	1	1.68	(0.06–10.05)	1.6
*Four years apart*					
2012 to 2016	1076	0	0.00	(0.00–0.43)	0.8
*Five years apart*					
2012 to 2017	935	3	0.42	(0.09–1.23)	0.8
*Six years apart*					
2012 to 2018	759	1	0.44	(0.01–2.75)	0.8
*Seven years apart*					
2012 to 2019	637	2	0.96	(0.13–3.91)	0.8
B/Yamagata					
*Two years apart*					
2017 to 2019	1585	2	0.60	(0.09–2.10)	0.6
*Three years apart*					
2014 to 2017	1326	4	0.74	(0.21–1.92)	0.6
*Five years apart*					
2014 to 2019	1018	1	0.11	(0.00–0.65)	0.6

*All models were adjusted for the age and sex of participants.

We observed limited seasons of B/Yamagata lineage circulation during the study period, so comparisons were only available between 2014, 2017, and 2019 seasons. Of these, significant protection was only observed between the 2014 and 2019 seasons (aOR: 0.11, CI: 0.00–0.65) [Table 2, Fig. 3]. When stratified by age, this protection remained for children ≤4 but was not significant for older children [Table S9]. There were minimal clade changes during the study time period, with no antigenic distances >0.6 on the log_2_ scale [Fig. 3 and S2]. Overall, we did not find the same pattern of protection from repeat infections among children from B/Yamagata as we observed for circulating subtypes of influenza A or B/Victoria. All results for Influenza B were similar in the sensitivity analyses conducted [Tables S10–S13].

## Discussion

Using RT-PCR-confirmed influenza infections and sequencing data from 2011 to 2019, we found that children infected with influenza A/H1N1pdm, A/H3N2, or B/Victoria were strongly protected from infection in subsequent seasons. Our findings regarding patterns of clade drift and sequence change in Nicaragua generally align with prior observations of influenza’s global circulation, where influenza A/H1N1pdm and B viruses have been observed to have less seasonal drift with slower rates of antigenic evolution and fewer epidemics as compared to influenza A/H3N2 viruses^27^. Additionally, these results support prior findings that repeat influenza infections can differentially boost antibody and immune responses depending upon those strains encountered early in life^28–32^. Overall, we found that protection waned with time and greater antigenic distance.

When we examined age-stratified results for A/H1N1pdm infection, we found that children aged ≥5 at infection experienced stronger protection than younger children. This accords with prior analyses showing that due to the slower rate of antigenic evolution in A/H1N1pdm, older children and adults are less susceptible to reinfection than young children^32,33^. This lower susceptibility possibly results from a broader level of immunity due to multiple exposures to A/H1N1pdm across multiple seasons; alternatively, this heightened protection may simply be the result of immune maturation or may be attributable to a broader spectrum of prior exposure to both H1 and H3 among older children who typically have a wider and more varied immune repertoire.

While we observed similar patterns in A/H3N2 repeat infections, these results were more heavily dependent on the level of antigenic change between seasons. Such findings align with antigenic cartography studies demonstrating that A/H3N2 generally evolves faster and with more punctuated evolution than A/H1N1pdm and B lineages^34^. The A/H3N2 3c2.A clade that emerged in 2014 is unique, as it possesses a glycosylation site that protects an important target of neutralizing antibodies. This clade has continued to circulate and has dominated influenza seasons despite individuals being repeatedly infected and acquiring some level of immunity^35^. Despite this, we observed protection from 2013 to 2014 when seasonal influenza vaccine effectiveness was low. Protection against circulating strains wanes faster against A/H3N2 than A/H1N1pdm^19^, which may explain why the period of homotypic protection observed here tended to be short, even after accounting for antigenic distances between dominant clades. Children aged ≥5 were generally better protected from repeat infection with A/H3N2, although children ≤4 infected in 2012 displayed a long duration of protection. Early life exposure to A/H3N2 significantly affects anti-3c2.A antibodies, so under-5 children infected in 2012 may have had different exposure patterns than older children, thereby driving an age disparity across season comparisons^35^.

B/Yamagata tends to circulate more frequently in adults and exhibit greater genetic diversity than B/Victoria^36,37^. B/Victoria tends to infect younger individuals than B/Yamagata, which has been hypothesized to result from differences in the age-varying prevalence of receptor-binding structures^38^. Alternatively, the declining prevalence of B/Victoria infection with age has been attributed to strengthened immunity over time due to accumulated immunity and lower levels of genetic change^36,37^. In general, we observed protection from repeated infections with B/Victoria among children, aligning with previously observed patterns of protection, as children appear to develop immunity over time resulting in strong protection by adulthood. By comparison, we saw very little protection from repeat infection with B/Yamagata, indicating that children may not develop durable protection in response to B/Yamagata infection.

This study is strengthened by our ability to study naturally occurring influenza infections in a population with high numbers of RT-PCR-confirmed positives. Influenza vaccination in Nicaragua is not common, with ~5% of our study population ever being vaccinated against influenza. Within this study, only symptomatic individuals who met testing criteria were RT-PCR tested, meaning that this analysis examined the effects of repeated symptomatic infection and excluded asymptomatic individuals. As we were primarily interested in the individual health effects of infection, we viewed this as an appropriate population. Because of the testing criteria for inclusion, we may have missed milder infections, which could potentially have biased our findings as the impacts of asymptomatic and mild infections could not be assessed by this study. While our main interest was in the role of symptomatic infections, we acknowledge that asymptomatic infections not captured by this study may have distorted the degree of homotypic protection found here by boosting immunity in between comparison seasons. Additionally, because the cohort used only includes children aged 14 or younger, our findings may not generalize to older populations. However, children are a highly vulnerable population for influenza infection and are major drivers of transmission; thus, we believe these findings have significant public health implications and are broadly generalizable to other pediatric populations, particularly those with low levels of annual influenza vaccination. One additional limitation is that we were unable to adjust in our analysis for the size of the influenza outbreak in each respective season; while we did limit our analysis for each subtype or lineage to those seasons where there was a clear peak, it is possible that some of the protection captured by this analysis was simply due to lack of exposure for certain individuals by chance due to lower viral circulation, rather than immunological protection. We do not believe that we would have observed the strength of protection noted in this paper simply due to lower circulation in certain seasons, particularly as the comparison group for each season was driven by the overall viral prevalence, but acknowledge that confidence intervals tended to be broader in seasons with smaller influenza epidemics.

The presence of homotypic protection against symptomatic infection between seasons of naturally occurring influenza has been explored prior to this study, but not in a setting with many RT-PCR-confirmed infections. Overall, we found that individuals infected with one influenza subtype or lineage had significantly lower odds of being infected with that same subtype or lineage in a subsequent season. These findings show that establishing individuals’ prior patterns of influenza infections can predict their subsequent risk and contribute to better, more specific vaccine development.

## Methods

### Study procedures

The Nicaraguan Pediatric Influenza Cohort Study (NPICS) protocol has been described previously^39^. Briefly, NPICS includes children aged 0-14 years and is conducted in Managua, Nicaragua, at the Health Center Sócrates Flores Vivas (HCSFV). Clinical history, sociodemographic information, and household characteristics are collected at enrollment. Annual visits are performed to collect blood, height, and weight data, and to re-administer surveys. All data is initially collected and managed via OpenClinica (OpenClinica LLC, Waltham, MA, https://openclinica.com/), which has been adapted and validated for the NPICS study. This study received ethical approval from the institutional review boards at the Ministry of Health of Nicaragua and the University of Michigan. Written informed parental consent is obtained for all participants, and verbal assent is obtained for all children ≥6 years. Parents agree to bring participants to the HCSFV at the first indication of fever; respiratory samples are collected for children meeting the testing definition of feverishness or fever of ≥37.8 °C with a cough, runny nose, and/or sore throat or lower respiratory symptoms.

Between January 2011 and December 2019, participants were followed from enrollment to their exit date or through 2019; additional children were enrolled each year to account for those aging out of the study and occasional loss to follow-up [Fig. S1]. Confirmed, acute infections were categorized based on their influenza infection type (A or B) and subtype (H1N1pdm or H3N2) or lineage (Victoria or Yamagata).

Participants were included in the analysis if they were followed during the entirety of an influenza season. For the primary analysis, individuals with homotypic infections (same subtype/lineage) during intervening years between the exposure year and outcome year under consideration in a given model were excluded from the analysis. Sensitivity analyses included such individuals.

Influenza vaccination levels are historically quite low in the study population; while in certain seasons children aged 6 months to 2 years of age were prioritized for influenza vaccination by the government, influenza vaccinations at the health center are dependent upon donations and annual vaccination percentages within the cohort were never above 6%, with an overall yearly vaccination rate among all children in the cohort of 2.7% [Table S1]. There were no significant differences in vaccination rates between the two age groups under comparison in the study.

### Laboratory methods

Sample collection and storage details have been described^39^. For RT-PCR testing, RNA was extracted from nasal/oropharyngeal swabs using the QIAamp^®^ Viral RNA Mini Kit (Qiagen Corporation, Valencia, CA). Influenza A and B viruses were amplified and typed/subtyped/lineaged according to CDC protocols. In total there was 6 influenza A samples that could not be subtyped, and 50 influenza B samples without an identifiable lineage—these samples were subsequently excluded from all subtype/lineage-specific analyses.

### Outcomes

The primary outcome was laboratory-confirmed symptomatic influenza A or B infection, defined by a positive RT-PCR test. Symptomatic was defined as meeting the testing criteria of feverishness or fever of ≥37.8 °C with a cough, runny nose, and/or sore throat or lower respiratory symptoms.

### Statistical analyses

The effect of prior influenza infection on protection against subsequent, homotypic infection was assessed using logistic regression models to estimate adjusted odds ratios (aORs) and 95% credible intervals (CIs). The exposure was influenza infection in a prior season, and the outcome was an infection of the same subtype or lineage in a subsequent season, examining individual-level associations between participants enrolled in the study during the relevant comparison years. Individuals with influenza infections in the intervening seasons were dropped from consideration for models comparing seasons separated by more than a year in order to accurately account for the waning effects of immunity. All models were adjusted for both age and sex and were run in R. Other potential confounders such as household crowding and underlying illness were considered and modeled in a directed acyclic graph [Fig. S4]. Ultimately we decided not to incorporate these variables into the modeling as their overall biasing effect would have been to attenuate the results toward the null, meaning that the findings presented here represent a likely underestimate of the effect of homotypic protection. In models grouped by age (>4 years vs. ≤4 years), the subject’s age group was determined by age at the time of the prior infection—this stratification by age was based on patterns of immune system development in children and commonly-accepted breakpoints in the literature, as well as the age structure of the cohort. The number of repeat infections (NRI) was determined for each set of comparisons between influenza seasons. To address the absence of repeat infections for certain comparison years (that caused convergence issues with frequentist models), we used Bayesian logistic models with non-informative priors of *N* (mean = 0, variance = 1000) on the beta coefficients. All statistical analyses were conducted in R (version 4.0.2).

### Phylogenetic analyses and antigenic distances

The final sequence dataset consisted of HA sequences for 189 A/H1N1pdm viruses, 381 A/H3N2 viruses, 151 B/Victoria viruses, and 74 B/Victoria viruses collected from our studies in Nicaragua from January 1, 2011, to December 31, 2019, using clinical samples collected during that time. Sequences were aligned using mafft v7.475^40^ and assigned to clades based on defining amino acid positions using the align_clades.py script available from the seasonal influenza build of Nextstrain, with visual confirmation that viruses from the same clade clustered monophyletically using IQ-TREE 2.0.3^41^. Distributions of clade assignments by subtype/lineage and year are available in the supplement [Figs. S2 and S3].

To measure the antigenic distance between influenza HA clades within each lineage, we constructed time-resolved phylogenetic trees (mafft 7.475, IQ-TREE 2.0.3, and TreeTime 0.8.1) that evenly sampled strains across major geographic regions and each month between January 1, 2010 and January 1, 2020, with priority given to strains with available hemagglutination inhibition (HI) measurements. We assigned strains to major historical clades with Nextstrain (Augur 12.0.0, and Auspice 2.25.1). We selected corresponding HI measurements for these strains from data provided by the CDC, the Victorian Infectious Diseases Reference Laboratory, and previously described in ref. ^34^. With this approach, we selected 3015 strains and 6870 HI measurements for A/H3N2, 2894 strains and 4911 HI measurements for A/H1N1pdm, 2989 strains and 5087 HI measurements for B/Victoria, and 2964 strains and 4427 HI measurements for B/Yamagata.

We calculated log_2_ normalized antigenic distances between pairs of strains with HI measurements and estimated serum potencies with a titer substitution model^42^. We calculated the mean log_2_ antigenic distance between each reference serum and all test strains in each clade, adjusted by serum potency of the corresponding reference strain, and then calculated the mean log_2_ distance between reference and test clades.

### Reporting summary

Further information on research design is available in the Nature Research Reporting Summary linked to this article.

## Supplementary information

Supplementary Information

Reporting Summary

## Data Availability

The data used in this study are available under restricted access for human subjects protections, access can be obtained by contacting Aubree Gordon (gordonal@umich.edu). The raw data are protected and are not available due to data privacy laws. The processed data are available upon request to be shared with outside investigators following University of Michigan IRB approval. Accession codes for virus sequences are provided in Supplementary Note 1.

## Code availability

R code is available on GitHub (link).
