# Supplementary material for: Homotypic protection against influenza in a pediatric cohort in Managua, Nicaragua
Source: Nat Commun. 2022 Mar 4;13:1190. doi: 10.1038/s41467-022-28858-9 (PMC8897407; doi:10.1038/s41467-022-28858-9)
Supplement: Supplementary file 1 — Supplementary Information [file 41467_2022_28858_MOESM1_ESM.pdf]

Homotypic protection against influenza in a pediatric cohort in Managua, Nicaragua

Table of Contents

Figures **2**

Figure S1. Flowchart of Study Participation **2**

Figure S2. Antigenic Distances **3**

Figure S3. Clade distributions by year and subtype/lineage **4**

Figure S4. Study DAG **5**

Tables **6**

Table S1. Influenza infections and vaccinations by season and type/subtype **6**

Table S2. H1N1 Repeat Infection Odds, Age-Stratified **7**

Table S3. H3N2 Repeat Infection Odds, Age-Stratified **8**

Table S4. H1N1 Repeat Infection Odds, Cumulative, Sensitivity Analysis **10**

Table S5. H1N1 Repeat Infection Odds, Age-Stratified, Sensitivity Analysis **11**

Table S6. H3N2 Repeat Infection Odds, Cumulative, Sensitivity Analysis **12**

Table S7. H3N2 Repeat Infection Odds, Age-Stratified, Sensitivity Analysis **13**

Table S8. Influenza B Victoria Repeat Infection Odds, Age-Stratified **15**

Table S9. Influenza B Yamagata Repeat Infection Odds, Age-Stratified **17**

Table S10. Influenza B Victoria Repeat Infection Odds, Cumulative, Sensitivity Analysis **18**

Table S11. Influenza B Victoria Repeat Infection Odds, Age-Stratified, Sensitivity Analysis **19**

Table S12. Influenza B Yamagata Repeat Infection Odds, Cumulative, Sensitivity Analysis **20**

Table S13. Influenza B Yamagata Repeat Infection Odds, Age-Stratified, Sensitivity Analysis **21**

Supplementary Note 1 .... **22**

Accession codes **22**

Figures

Figure S1. Flowchart of Study Participation

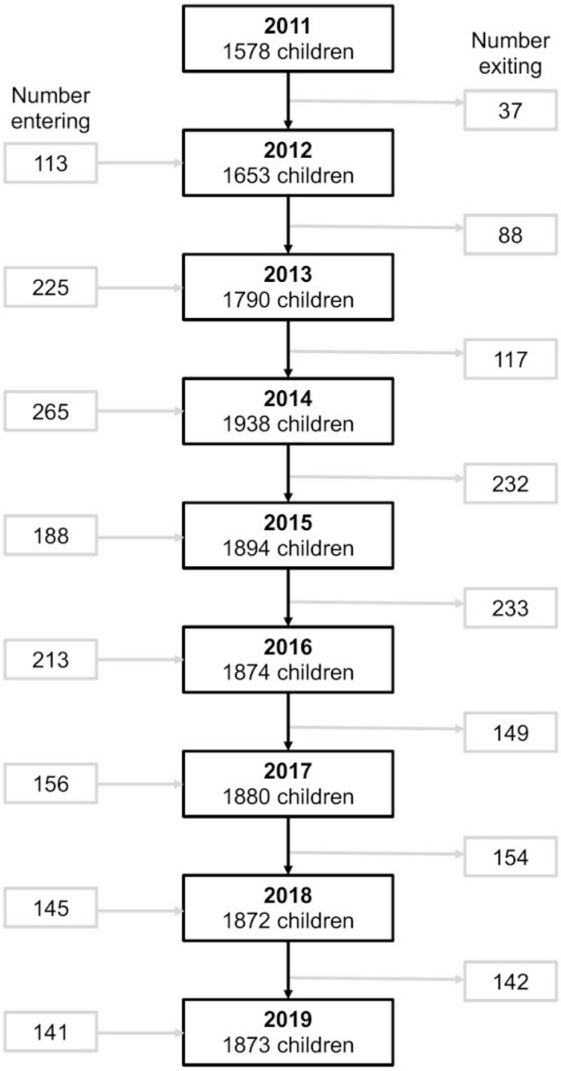

Figure S2. Antigenic Distances

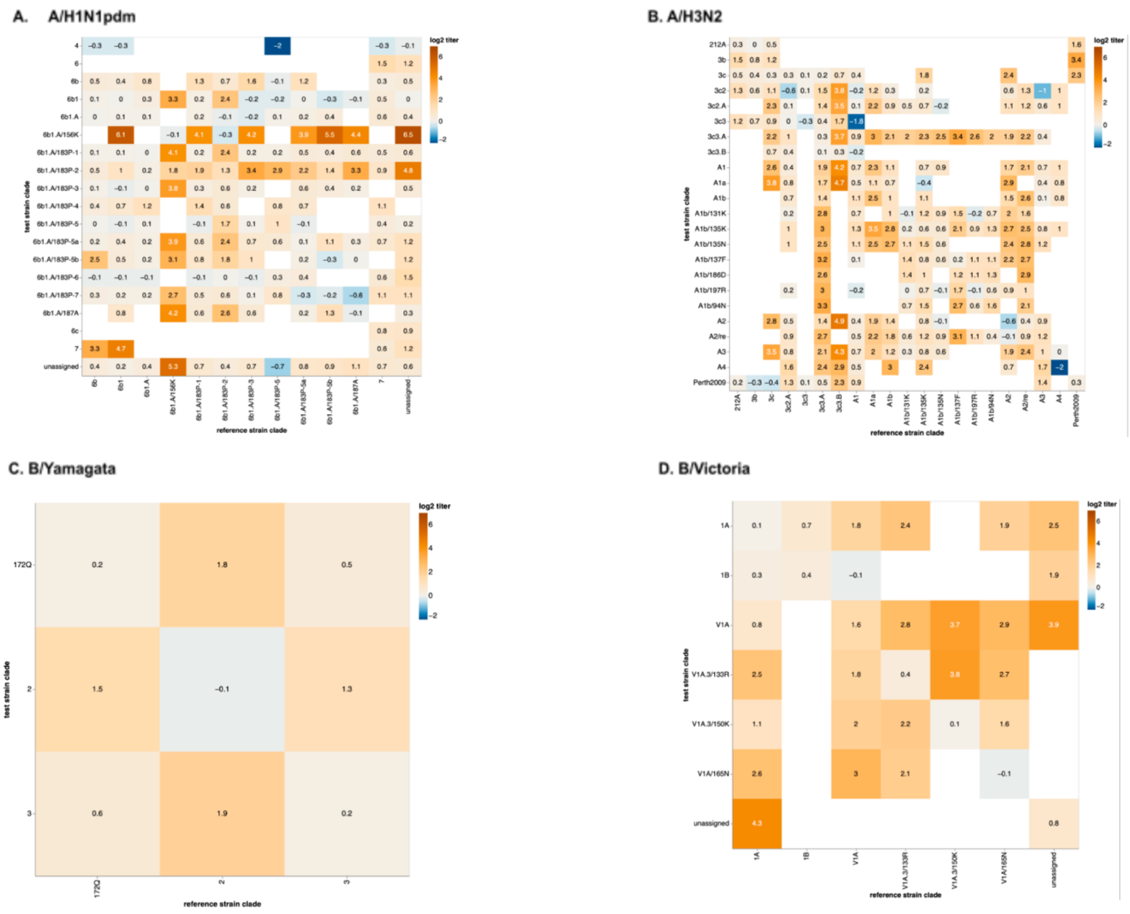

Figure legend: A. Heatmap depicting the antigenic distances between globally circulating clades of A/H1N1pdm between 2011 and 2019. B. Heatmap depicting the antigenic distances between globally circulating clades of A/H3N2 between 2011 and 2019. C. Heatmap depicting the antigenic distances between globally circulating clades of B/Yamagata between 2011 and 2019. D. Heatmap depicting the antigenic distances between globally circulating clades of B/Victoria between 2011 and 2019.

Figure S3. Clade distributions by year and subtype/lineage

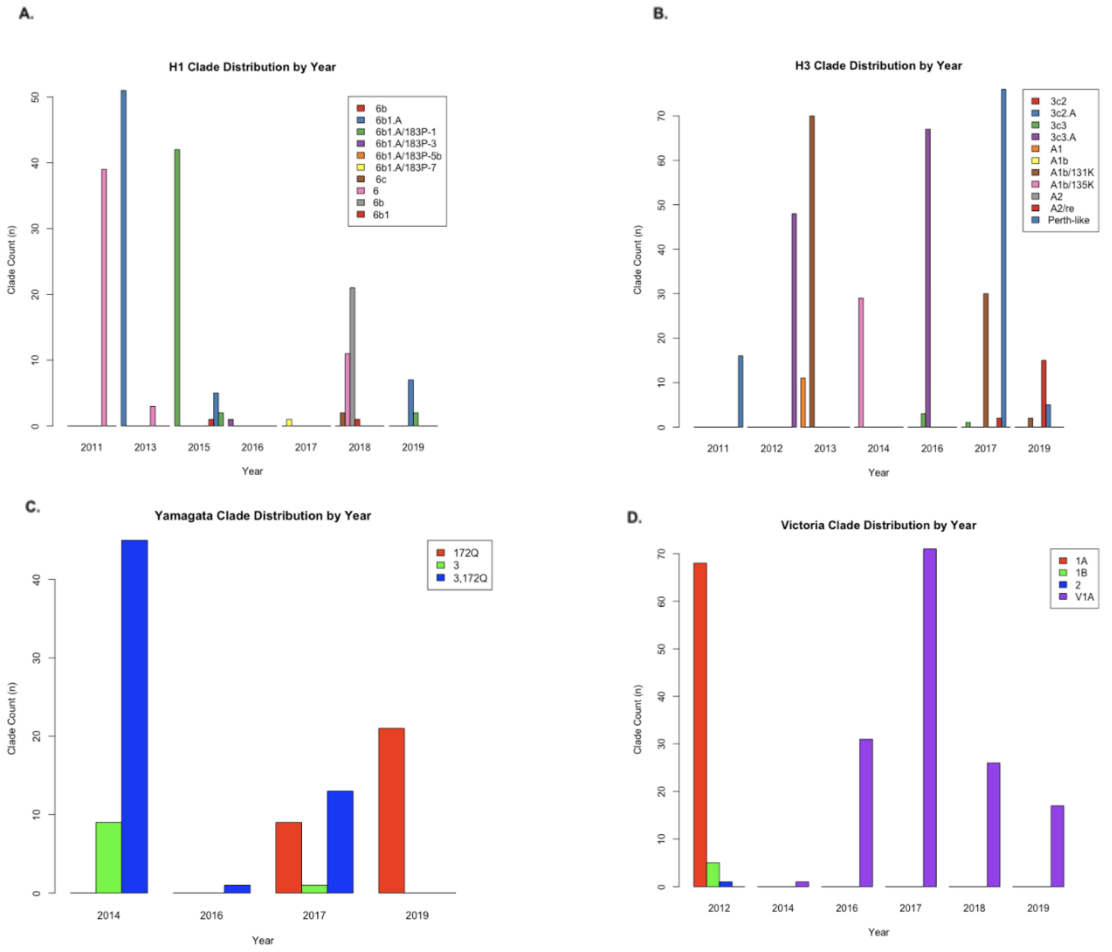

Figure Legend: A. Counts of clade distributions by year for sequencing data for all H1 viruses from Nicaragua during the study time period. B. Counts of clade distributions by year for sequencing data for all H3 viruses from Nicaragua during the study time period. C. Counts of clade distributions by year for sequencing data for all Yamagata viruses from Nicaragua during the study time period. D. Counts of clade distributions by year for sequencing data for all Victoria viruses from Nicaragua during the study time period.

Figure S4. Study DAG

Homotypic Analysis DAG

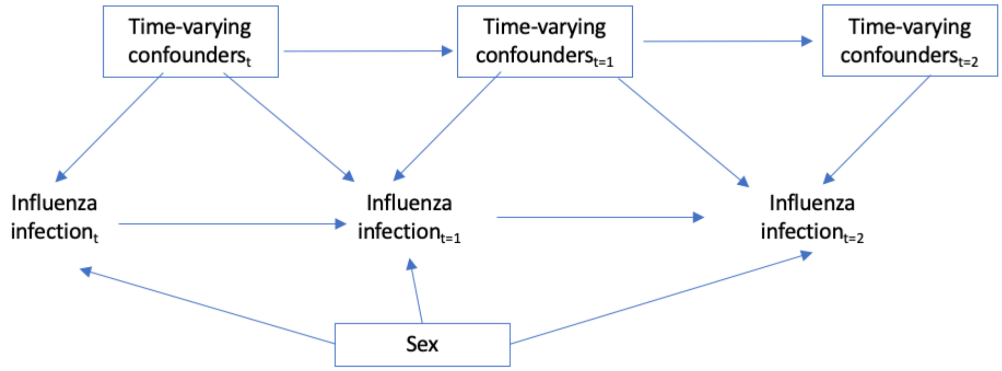

*Time-varying confounders considered for this study: age (controlled), healthcare-seeking behavior, household crowding, underlying health issues

Tables

Table S1. Influenza infections and vaccinations by season and type/subtype

		**H1N1**		**H3N2**		**IB**		**Vaccinated**	
Influenza Season	N	n	%	n	%	n	%	n	
2011	1,578	102	6.5	16	1.0	2	0.1	10	0.6
2012	1,653	0	0.0	49	3.0	154	9.3	69	4.2
2013	1,790	66	3.7	159	8.9	2	0.1	38	2.1
2014	1,938	0	0.0	168	8.7	205	10.6	48	2.5
2015	1,894	153	8.1	10	0.5	0	0.0	93	4.9
2016	1,874	1	0.1	134	7.2	31	1.7	20	1.1
2017	1,880	1	0.1	150	8.0	220	11.7	25	1.3
2018	1,872	198	10.6	0	0.0	47	2.5	27	1.4
2019	1,873	21	1.2	181	9.7	137	7.3	111	5.9

*In most years, the influenza season in Nicaragua is entirely contained within a single calendar year, however occasionally cases will continue into the next year

Table S2. H1N1 Repeat Infection Odds, Age-Stratified

Seasons	N	NRI	OR	95% CI
**4 and under**				
Two years apart				
Summary		1	0.12	(0.02-0.85)
2011 to 2013	503	1	0.23	(0.01-1.32)
2013 to 2015	501	0	0.00	(0.00-0.06)
Three years apart				
2015 to 2018	550	3	0.21	(0.05-0.61)
Four years apart				
2011 to 2015	408	1	0.12	(0.00-0.69)
Five years apart				
2013 to 2018	379	1	0.16	(0.01, 0.92)
Seven years apart				
2011 to 2018	330	3	0.71	(0.15-2.24)
**5 and over**				
Two years apart				
Summary		0	0.00	(0.00-0.05)
2011 to 2013	965	0	0.00	(0.00-0.27)
2013 to 2015	955	0	0.00	(0.00-0.23)
Three years apart				
2015 to 2018	814	0	0.00	(0.00-0.09)
Four years apart				
2011 to 2015	730	0	0.00	(0.00-0.22)
Five years apart				
2013 to 2018	506	1	0.74	(0.03-4.50)
Seven years apart				
2011 to 2018	329	0	0.00	(0.00-0.22)

Table S3. H3N2 Repeat Infection Odds, Age-Stratified

Seasons	N	NRI	OR	95% CI
**4 and under**				
One year apart				
Summary		11	0.37	(0.20-0.70)
2012 to 2013	489	2	0.45	(0.06-1.69)
2013 to 2014	560	5	0.30	(0.10-0.70)
2016 to 2017	583	4	0.48	(0.09-1.56)
Two years apart				
Summary		10	0.41	(0.21-0.80)
2012 to 2014	377	1	0.22	(0.01-1.31)
2014 to 2016	507	2	0.27	(0.04-0.99)
2017 to 2019	596	7	0.50	(0.20-1.06)
Three years apart				
Summary		16	0.77	(0.45-1.34)
Summary for post-2014		8	0.54	(0.25-1.14)
2013 to 2016	385	8	1.70	(0.67-3.96)
2014 to 2017	454	5	0.65	(0.21-1.62)
2016 to 2019	479	3	0.36	(0.08-1.05)
Four years apart				
2012 to 2016	267	0	0.00	(0.00-0.35)
2013 to 2017	347	7	1.81	(0.66-4.43)
Five years apart				
2012 to 2017	246	0	0.00	(0.00-0.33)
2014 to 2019	385	5	0.83	(0.27-2.10)
Six years apart				
2013 to 2019	302	5	1.06	(0.33-2.76)
Seven years apart				
2012 to 2019	217	0	0.00	(0.00-0.42)
**5 and over**				
One year apart				
Summary		2	0.20	(0.05-0.81)
2012 to 2013	1077	0	0.00	(0.00-0.15)
2013 to 2014	1115	0	0.00	(0.00-0.10)
2016 to 2017	1142	2	0.41	(0.06-1.56)
Two years apart				
Summary		2	0.19	(0.05-0.77)
2012 to 2014	958	0	0.00	(0.00-0.22)

2014 to 2016	957	0	0.00	(0.00-0.07)
2017 to 2019	990	2	0.47	(0.07-1.66)
Three years apart				
Summary		7	0.71	(0.32-1.54)
Summary for post-2014		3	0.42	(0.13-1.36)
2013 to 2016	720	4	1.34	(0.38-3.58)
2014 to 2017	773	1	0.21	(0.01-1.20)
2016 to 2019	830	2	0.46	(0.06-1.63)
Four years apart				
2012 to 2016	597	0	0.00	(0.00-0.26)
2013 to 2017	560	1	0.36	(0.01-2.08)
Five years apart				
2012 to 2017	452	2	3.05	(0.40-12.85)
2014 to 2019	516	3	0.73	(0.16-2.23)
Six years apart				
2013 to 2019	330	3	1.51	(0.32-4.97)
Seven years apart				
2012 to 2019	245	1	1.39	(0.05-9.88)

Table S4. H1N1 Repeat Infection Odds, Cumulative, Sensitivity Analysis

Seasons	N	NRI	OR	95% CI
Two years apart				
2011 to 2013	1468	1	0.20	(0.01-1.08)
2013 to 2015	1456	0	0.01	(0.00-0.07)
Three years apart				
2015 to 2018	1365	3	0.18	(0.04-0.51)
Four years apart				
2011 to 2015	1186	1	0.14	(0.00-0.77)
Five years apart				
2013 to 2018	972	2	0.41	(0.06-1.48)
Seven years apart				
2011 to 2018	747	3	0.64	(0.14-1.89)

Table S5. H1N1 Repeat Infection Odds, Age-Stratified, Sensitivity Analysis

Seasons	N	NRI	OR	95% CI
**4 and under**				
Two years apart				
2011 to 2013	503	1	0.23	(0.01-1.32)
2013 to 2015	501	0	0.00	(0.00-0.06)
Three years apart				
2015 to 2018	551	3	0.21	(0.05-0.61)
Four years apart				
2011 to 2015	434	1	0.13	(0.00-0.72)
Five years apart				
2013 to 2018	435	1	0.18	(0.01, 1.02)
Seven years apart				
2011 to 2018	392	3	0.80	(0.18-2.51)
**5 and over**				
Two years apart				
2011 to 2013	965	0	0.00	(0.00-0.27)
2013 to 2015	955	0	0.00	(0.00-0.23)
Three years apart				
2015 to 2018	814	0	0.00	(0.00-0.09)
Four years apart				
2011 to 2015	752	0	0.00	(0.00-0.23)
Five years apart				
2013 to 2018	537	1	0.78	(0.03, 4.89)
Seven years apart				
2011 to 2018	355	0	0.00	(0.00-0.32)

Table S6. H3N2 Repeat Infection Odds, Cumulative, Sensitivity Analysis

Seasons	N	NRI	OR	95% CI
One year apart				
2012 to 2013	1566	2	0.48	(0.06-1.41)
2013 to 2014	1675	5	0.32	(0.10-0.67)
2016 to 2017	1725	6	0.52	(0.19-1.05)
Two years apart				
2012 to 2014	1464	1	0.17	(0.01-0.94)
2014 to 2016	1474	2	0.16	(0.02-0.53)
2017 to 2019	1586	9	0.67	(0.29-1.22)
Three years apart				
2013 to 2016	1244	12	1.76	(0.88-3.28)
2014 to 2017	1328	6	0.62	(0.24-1.36)
2016 to 2019	1438	5	0.45	(0.15, 1.03)
Four years apart				
2012 to 2016	1076	0	0.00	(0.00-0.12)
2013 to 2017	1106	9	1.47	(0.65-2.95)
Five years apart				
2012 to 2017	945	2	1.19	(0.17-4.46)
2014 to 2019	1058	9	1.01	(0.45-2.00)
Six years apart				
2013 to 2019	849	8	1.07	(0.45-2.23)
Seven years apart				
2012 to 2019	699	2	1.15	(0.16-4.29)

Table S7. H3N2 Repeat Infection Odds, Age-Stratified, Sensitivity Analysis

Seasons	N	NRI	OR	95% CI
**4 and under**				
One year apart				
2012 to 2013	489	2	0.45	(0.06-1.69)
2013 to 2014	560	5	0.30	(0.10-0.70)
2016 to 2017	583	4	0.48	(0.09-1.56)
Two years apart				
2012 to 2014	454	1	0.23	(0.01-1.33)
2014 to 2016	511	2	0.27	(0.04-0.99)
2017 to 2019	596	7	0.50	(0.20-1.06)
Three years apart				
2013 to 2016	460	8	1.78	(0.70-4.05)
2014 to 2017	494	5	0.66	(0.22-1.62)
2016 to 2019	556	3	0.34	(0.08-0.99)
Four years apart				
2012 to 2016	387	0	0.00	(0.00-0.21)
2013 to 2017	448	8	1.83	(0.74-4.19)
Five years apart				
2012 to 2017	381	0	0.00	(0.00-0.40)
2014 to 2019	463	6	1.07	(0.38-2.54)
Six years apart				
2013 to 2019	419	5	0.84	(0.27-2.13)
Seven years apart				
2012 to 2019	356	1	0.80	(0.03-5.06)
**5 and over**				
One year apart				
2012 to 2013	1077	0	0.00	(0.00-0.15)
2013 to 2014	1115	0	0.00	(0.00-0.10)
2016 to 2017	1142	2	0.41	(0.06-1.56)
Two years apart				
2012 to 2014	1010	0	0.00	(0.00-0.18)
2014 to 2016	963	0	0.00	(0.00-0.06)
2017 to 2019	990	2	0.47	(0.07-1.66)
Three years apart				
2013 to 2016	784	4	1.47	(0.41-3.96)
2014 to 2017	834	1	0.22	(0.01-1.24)
2016 to 2019	882	2	0.47	(0.07-1.67)

Four years apart				
2012 to 2016	689	0	0.00	(0.00-0.33)
2013 to 2017	658	1	0.33	(0.01-1.95)
Five years apart				
2012 to 2017	564	2	2.87	(0.38-11.67)
2014 to 2019	595	3	0.76	(0.17-2.29)
Six years apart				
2013 to 2019	430	3	1.31	(0.28-4.17)
Seven years apart				
2012 to 2019	343	1	1.22	(0.04-8.10)

Table S8. Influenza B Victoria Repeat Infection Odds, Age-Stratified

Seasons	N	NRI	OR	95% CI
**4 and under**				
One year apart				
Summary		0	0.00	(0.00-0.28)
2016 to 2017	583	0	0.00	(0.00-0.26)
2017 to 2018	609	0	0.00	(0.00-0.29)
2018 to 2019	625	0	0.00	(0.00-1.23)
Two years apart				
Summary		0	0.00	(0.00-0.56)
2016 to 2018	510	0	0.00	(0.00-0.90)
2017 to 2019	585	0	0.00	(0.00-0.23)
Three years apart				
2016 to 2019	483	0	0.00	(0.00-1.38)
Four years apart				
2012 to 2016	387	0	0.00	(0.00-0.32)
Five years apart				
2012 to 2017	373	1	0.17	(0.01-0.95)
Six years apart				
2012 to 2018	330	1	0.58	(0.02-3.96)
Seven years apart				
2012 to 2019	312	1	0.65	(0.02-4.54)
**5 and over**				
One year apart				
Summary		0	0.00	(0.00-0.25)
2016 to 2017	1142	0	0.00	(0.00-0.40)
2017 to 2018	1118	0	0.00	(0.00-0.22)
2018 to 2019	1105	0	0.00	(0.00-0.30)
Two years apart				
Summary		0	0.00	(0.00-0.68)
2016 to 2018	947	0	0.00	(0.00-1.15)
2017 to 2019	965	0	0.00	(0.00-0.31)
Three years apart				

2016 to 2019	812	1	3.50	(0.13-23.37)
Four years apart				
2012 to 2016	689	0	0.00	(0.00-2.68)
Five years apart				
2012 to 2017	562	2	0.72	(0.10-2.74)
Six years apart				
2012 to 2018	429	0	0.00	(0.00-0.79)
Seven years apart				
2012 to 2019	325	1	1.16	(0.04-9.13)

Table S9. Influenza B Yamagata Repeat Infection Odds, Age-Stratified

Seasons	N	NRI	OR	95% CI
**Four and under**				
Two years apart				
2017 to 2019	596	1	0.63	(0.02-3.80)
Three years apart				
2014 to 2017	494	2	0.94	(0.13-3.78)
Five years apart				
2014 to 2019	447	0	0.00	(0.00-0.09)
**Five and over**				
Two years apart				
2017 to 2019	989	1	0.46	(0.02-2.65)
Three years apart				
2014 to 2017	832	2	0.53	(0.08-1.96)
Five years apart				
2014 to 2019	571	1	0.31	(0.01-1.88)

Table S10. Influenza B Victoria Repeat Infection Odds, Cumulative, Sensitivity Analysis

Seasons	N	NRI	OR	95% CI
One year apart				
2016 to 2017	1725	0	0.00	(0.00-0.18)
2017 to 2018	1727	0	0.00	(0.00-0.21)
2018 to 2019	1730	0	0.00	(0.00-0.41)
Two years apart				
2016 to 2018	1573	0	0.00	(0.00-0.40)
2017 to 2019	1586	0	0.00	(0.00-0.21)
Three years apart				
2016 to 2019	1438	1	1.85	(0.06-11.35)
Four years apart				
2012 to 2016	1076	0	0.00	(0.00-0.43)
Five years apart				
2012 to 2017	945	3	0.43	(0.10-1.24)
Six years apart				
2012 to 2018	816	1	0.44	(0.02-2.62)
Seven years apart				
2012 to 2019	699	2	1.02	(0.14-4.15)

Table S11. Influenza B Victoria Repeat Infection Odds, Age-Stratified, Sensitivity Analysis

Seasons	N	NRI	OR	95% CI
**4 and under**				
One year apart				
2016 to 2017	583	0	0.00	(0.00-0.26)
2017 to 2018	609	0	0.00	(0.00-0.29)
2018 to 2019	625	0	0.00	(0.00-1.23)
Two years apart				
2016 to 2018	566	0	0.00	(0.00-0.87)
2017 to 2019	596	0	0.00	(0.00-0.32)
Three years apart				
2016 to 2019	556	0	0.00	(0.00-1.65)
Four years apart				
2012 to 2016	387	0	0.00	(0.00-0.32)
Five years apart				
2012 to 2017	381	1	0.17	(0.01-0.99)
Six years apart				
2012 to 2018	367	1	0.65	(0.02-4.38)
Seven years apart				
2012 to 2019	356	1	0.71	(0.03-4.96)
**5 and over**				
One year apart				
2016 to 2017	1142	0	0.00	(0.00-0.40)
2017 to 2018	1118	0	0.00	(0.00-0.22)
2018 to 2019	1105	0	0.00	(0.00-0.30)
Two years apart				
2016 to 2018	1007	0	0.00	(0.00-1.36)
2017 to 2019	990	0	0.00	(0.00-0.30)
Three years apart				
2016 to 2019	882	0	0.00	(0.00-1.89)
Four years apart				
2012 to 2016	689	0	0.00	(0.00-2.68)
Five years apart				
2012 to 2017	564	2	0.74	(0.10-2.79)
Six years apart				
2012 to 2018	449	0	0.00	(0.00-0.69)
Seven years apart				
2012 to 2019	343	1	1.17	(0.04-9.27)

Table S12. Influenza B Yamagata Repeat Infection Odds, Cumulative, Sensitivity Analysis

Seasons	N	NRI	OR	95% CI
Two years apart				
2017 to 2019	1586	2	0.60	(0.08-2.10)
Three years apart				
2014 to 2017	1328	4	0.72	(0.21-1.87)
Five years apart				
2014 to 2019	1058	1	0.12	(0.00-0.68)

Table S13. Influenza B Yamagata Repeat Infection Odds, Age-Stratified, Sensitivity Analysis

Seasons	N	NRI	OR	95% CI
**Four and under**				
Two years apart				
2017 to 2019	596	1	0.63	(0.02-3.80)
Three years apart				
2014 to 2017	494	2	0.94	(0.13-3.78)
Five years apart				
2014 to 2019	463	0	0.00	(0.00-0.09)
**Five and over**				
Two years apart				
2017 to 2019	990	1	0.47	(0.02-2.65)
Three years apart				
2014 to 2017	834	2	0.52	(0.08-1.89)
Five years apart				
2014 to 2019	595	1	0.33	(0.01-1.94)

Supplementary Note 1

Accession codes

AHZ36884
AHZ36906
AHZ36895
AHZ36928
AHZ36917
AHZ36708
AHZ36697
QCT23392
AUL82501
AHZ36741
AHZ36785
AHZ36752
QCT22848
AHZ36774
QCT23317
QCT23306
AHZ36961
QCT23370
QCT23414
AHZ36818
AHZ36972
QCT23513
AHZ36862
QCT23535
AUL82512
AHZ36851
AHZ36873
AHZ36719
AHZ36730
AHZ36763
AHZ36796
AHZ36807
QCT22870
AHZ36939
AHZ36950
AHZ36829

AHZ36840
QCT22828
QCT23229
QCT22947
AHZ37016
QCT23130
AHZ37027
AUL82322
QCT23108
QCT22967
QCT23590
AHZ37038
AHZ37049
AHZ37071
AUL82567
QCT23174
AHZ37060
QCT22839
QCT23273
QCT23044
QCT23163
AIL95120
AIL95109
AHZ37082
AHZ37093
AUL82380
AUL82523
AHZ37104
AUL82545
AUL82534
AUL82556
AHZ36994
AUL82578
AUL82589
AUL82600
AUL82611
AUL82391
QCT23077
QCU44812
QCT23066

QCT22881
QCT23436
ASZ79121
ASZ79168
ASZ79212
ASZ79179
QCT23403
QCT23601
QCT23088
ASZ79223
QCT23141
QCT23502
QCT23055
QCT23119
QCT23579
QCT23612
AUL82622
QCT23491
QCT23623
QCT22925
QCT23469
AUL82402
QCT23000
ASZ79157
AUL82633
QCU45038
QCU45008
QCU45090
QCU45050
QCU44831
QCU44924
ASZ79122
QCU44790
QCU45199
QCU44944
QCU45080
ASZ79112
QCU45061
QCT23359
QCT23251

QCT23033
ASZ79190
QCT23152
QCT23546
QCT23524
ASZ79101
ASZ79201
QCT23634
AUL82413
QCT23097
QCT23262
QCT22903
AUL82311
QCT23568
QCT23295
QCU44976
QCU44889
AUL82490
QCT22978
QCT23011
AUL82468
QCT23557
QCT22914
QCT22817
AUL82457
AUL82446
AUL82300
QCT23218
QCT23480
AUL82479
QCT22989
AUL82289
AUL82435
QCT23284
ASZ79311
ASZ79267
ASZ79278
QCT22936
ASZ79245
ASZ79300

ASZ79234
AUL82278
ASZ79289
ASZ79256
ASZ79148
AUL82267
ASZ79139
AUL82424
QCU44743
QCT23339
QCT23207
QCT23196
QCT23645
QCT22958
QCU44899
QCT23350
QCT23381
QCU45189
QCU44822
QCT22892
QCU44910
QCT23447
QCU44728
QCT23022
QCT23656
QCT23240
QCU45121
QCU44998
QCU44881
QCU44954
QCU45220
QCU44717
QCT23458
QCU45019
QCU45028
QCU44738
QCU45209
QCU45111
QCU45132
QCT23185

QCT23667
QCT22859
QCT23425
QCU44869
QCU45159
QCU45242
QCU44836
QCU44847
QCU45069
QCU44781
QCU44986
QCU44965
QCU44992
QCU44858
QCU44707
QCU45139
QCU44935
QCU44802
QCU44751
QCU45170
QCU45102
QCU45179
QCU44760
QCU45231
QCU44770
QCU45148
QCU45252
QCU45262
