# Supplementary material for: Homotypic protection against influenza in a pediatric cohort in Managua, Nicaragua
Source: Nat Commun. 2022 Mar 4;13:1190. doi: 10.1038/s41467-022-28858-9 (PMC8897407; doi:10.1038/s41467-022-28858-9)
Supplement: Supplementary file 2 — Reporting Summary [file 41467_2022_28858_MOESM2_ESM.pdf]

nature portfolio

Corresponding author(s): Aubree Gordon

Last updated by author(s): Jan 27, 2022

Reporting Summary

Nature Portfolio wishes to improve the reproducibility of the work that we publish. This form provides structure for consistency and transparency in reporting. For further information on Nature Portfolio policies, see our Editorial Policies and the Editorial Policy Checklist.

Statistics

For all statistical analyses, confirm that the following items are present in the figure legend, table legend, main text, or Methods section.

n/a	Confirmed	
☐	☒	The exact sample size (*n*) for each experimental group/condition, given as a discrete number and unit of measurement
☐	☒	A statement on whether measurements were taken from distinct samples or whether the same sample was measured repeatedly
☒	☐	The statistical test(s) used AND whether they are one- or two-sided<br>*Only common tests should be described solely by name; describe more complex techniques in the Methods section.*
☐	☒	A description of all covariates tested
☐	☒	A description of any assumptions or corrections, such as tests of normality and adjustment for multiple comparisons
☐	☒	A full description of the statistical parameters including central tendency (e.g. means) or other basic estimates (e.g. regression coefficient) AND variation (e.g. standard deviation) or associated estimates of uncertainty (e.g. confidence intervals)
☒	☐	For null hypothesis testing, the test statistic (e.g. *F*, *t*, *r*) with confidence intervals, effect sizes, degrees of freedom and *P* value noted<br>*Give P values as exact values whenever suitable.*
☐	☒	For Bayesian analysis, information on the choice of priors and Markov chain Monte Carlo settings
☐	☒	For hierarchical and complex designs, identification of the appropriate level for tests and full reporting of outcomes
☒	☐	Estimates of effect sizes (e.g. Cohen's *d*, Pearson's *r*), indicating how they were calculated

Our web collection on statistics for biologists contains articles on many of the points above.

Software and code

Policy information about availability of computer code

Data collection	Numerous tools are implemented to manage the large amounts of data involved with study operation, providing real-time monitoring and facilitating management of screening processes, registration, and long-term follow-up [12]. These tools are highly flexible and secure, and enable compliance with GCP and regulatory guidelines, including US federal regulations (21 CFR 11), via differentiated user roles and privileges, password and user authentication security, and comprehensive auditing to record and monitor access and data changes. The components of the custom informatics system are presented in Additional file 1: Table S1. OpenClinica is the main tool used for data management (OpenClinica LLC, Waltham, MA, https://openclinica.com/). OpenClinica was adapted for the NPICS study and validated to ensure accuracy, reliability, consistent performance, and an audit trail to track invalid/altered records. The system produces accurate paper copies of electronic records via data exports. Backup and restore processes are documented and validated. As many data and lab results as possible are entered automatically through tablets, smart phones, barcodes, and electronic transfer to study databases. When direct entry of data is used, the software uses prompts for out-of-range data and consistent terminology use. Data collection/entry is supervised, and double data entry is used, with multiple “check functions” in place. Alteration of data is strictly controlled.
Data analysis	Data was analyzed using R for all modeling including Bayesian modeling (version 4.0.2). Phylogenetic analyses were conducted using mafft v7.475, IQ-TREE 2.0.3, and TreeTime 0.8.1.

For manuscripts utilizing custom algorithms or software that are central to the research but not yet described in published literature, software must be made available to editors and reviewers. We strongly encourage code deposition in a community repository (e.g. GitHub). See the Nature Portfolio guidelines for submitting code & software for further information.

Data

Policy information about availability of data

All manuscripts must include a data availability statement. This statement should provide the following information, where applicable:

- Accession codes, unique identifiers, or web links for publicly available datasets
- A description of any restrictions on data availability
- For clinical datasets or third party data, please ensure that the statement adheres to our policy

The data used in this study are available under restricted access for human subjects protections, access can be obtained by contacting Aubree Gordon (gordonal@umich.edu). The raw data are protected and are not available due to data privacy laws. The processed data are available upon request to be shared with outside investigators following University of Michigan IRB approval.

Field-specific reporting

Please select the one below that is the best fit for your research. If you are not sure, read the appropriate sections before making your selection.

☐ Life sciences ☒ Behavioural & social sciences ☐ Ecological, evolutionary & environmental sciences

For a reference copy of the document with all sections, see nature.com/documents/nr-reporting-summary-flat.pdf

Behavioural & social sciences study design

All studies must disclose on these points even when the disclosure is negative.

Study description	Data for this study was collected from a prospective cohort study of children in Nicaragua; study enrollment began in 2011 and continued through 2019 with children exiting the study as they aged out and new children being enrolled; all data used in this study was quantitative.
Research sample	The research sample was children aged 0-14 in District II of Managua, Nicaragua. This sample is appropriate for assessing the impact of influenza among children in a tropical, low income setting. The study population was balanced on sex and was sampled in order to be representative of the overall population of children residing in District 2 of Managua.
Sampling strategy	To maximize follow-up time of participants and thus information on infection history, initial enrollment of three- to eleven-year-old children was done through a random selection of all participants from our previous influenza cohort study. Additional children ≤2 years were recruited for NPICS through house-to-house visits. Each month, newborn infants aged ≤4 weeks are enrolled into the NPICS. Full sample size selection and rationale are detailed in the original study outline paper (cited in the manuscript); briefly, while no specific power calculations were conducted for this exact analysis, the very high enrollment, low attrition of participants, and high number of influenza positives lead to us being very confident in the power of this study and the sufficiency of the sample sizes.
Data collection	Each March/April, annual surveys are conducted and a blood sample is collected, before the influenza season begins, typically in June. Annual surveys include a socio-economic and household risk factor questionnaire, a breastfeeding questionnaire, height and weight, and a satisfaction questionnaire. Survey data are collected directly into a tablet computer or smart phone, with home factors (i.e., variables that are constant between household members) collected separately from participant-specific factors. Blood samples of 7 ml are collected from cohort participants aged ≥2 years and 3 ml from children ≥6 months and <2 years. Parents of participants agreed to bring their child(ren) in to the HCSFV at the first sign of fever. Upon presentation at the HCSFV, participants identify themselves in the reception area. All participants have a fingerprint scan on file and are issued a barcoded ID card. Confirmation of study participation is performed by scanning either the participant's study ID card or fingerprint. The participant is then directed to the study area, where a study nurse measures and records their height, weight, and tympanic temperature. Next, the child is examined by a study physician, who records information systematically on the history of illness and current symptoms. Data on >80 variables are collected, including a second tympanic temperature, blood pressure, respiratory and heart rates, lower and upper respiratory symptoms, gastrointestinal symptoms, and nutritional status. Researchers involved in all data collection and testing for the study were blinded to the study hypothesis explored in this analysis.
Timing	Data collection began in January 2011 and continued through December 2019.
Data exclusions	No data were excluded from the overall study analysis; for certain comparisons individuals who did not participate in the study for that season were not included, as detailed in the methods section.
Non-participation	Between 37 to 233 exited the study each year; between 113-265 children entered the study each year. Most children exited the study due to aging out (turning 15 years of age) rather than due to non-response.
Randomization	Participants were not allocated into different groups.

Reporting for specific materials, systems and methods

We require information from authors about some types of materials, experimental systems and methods used in many studies. Here, indicate whether each material, system or method listed is relevant to your study. If you are not sure if a list item applies to your research, read the appropriate section before selecting a response.

Materials & experimental systems

n/a	Involved in the study
☒	☐ Antibodies
☒	☐ Eukaryotic cell lines
☒	☐ Palaeontology and archaeology
☒	☐ Animals and other organisms
☐	☒ Human research participants
☒	☐ Clinical data
☒	☐ Dual use research of concern

Methods

n/a	Involved in the study
☒	☐ ChIP-seq
☒	☐ Flow cytometry
☒	☐ MRI-based neuroimaging

Human research participants

Policy information about studies involving human research participants

Population characteristics	See above.
Recruitment	See above. We do not believe that self-selection bias is a significant concern for this study nor that it would influence our findings; there is no financial compensation for participation and the provision of health care mainly serves simply to encourage regular clinic attendance when exhibiting symptoms to ensure adequate capture of influenza episodes.
Ethics oversight	This study received ethical approval from the institutional review boards at the Ministry of Health of Nicaragua and the University of Michigan

Note that full information on the approval of the study protocol must also be provided in the manuscript.
